# Recombinant Brain Natriuretic Peptide for the Prevention of Contrast-Induced Nephropathy in Patients with Chronic Kidney Disease Undergoing Nonemergent Percutaneous Coronary Intervention or Coronary Angiography: A Randomized Controlled Trial

**DOI:** 10.1155/2016/5985327

**Published:** 2016-02-02

**Authors:** Jinming Liu, Yanan Xie, Fang He, Zihan Gao, Yuming Hao, Xiuguang Zu, Liang Chang, Yongjun Li

**Affiliations:** ^1^Department of Cardiology, Second Hospital, Hebei Medical University, Shijiazhuang, Hebei 050000, China; ^2^Department of Cardiology, Weixian Hospital of Traditional Chinese Medicine, Xingtai, Hebei 054700, China

## Abstract

The role of brain natriuretic peptide (BNP) in the prevention of contrast-induced nephropathy (CIN) is unknown. This study aimed to investigate BNP's effect on CIN in chronic kidney disease (CKD) patients undergoing elective percutaneous coronary intervention (PCI) or coronary angiography (CAG). The patients were randomized to BNP (0.005 *μ*g/kg/min before contrast media (CM) exposure and saline hydration, *n* = 106) or saline hydration alone (*n* = 103). Cystatin C, serum creatinine (SCr) levels, and estimated glomerular filtration rates (eGFR) were assessed at several time points. The primary endpoint was CIN incidence; secondary endpoint included changes in cystatin C, SCr, and eGFR. CIN incidence was significantly lower in the BNP group compared to controls (6.6% versus 16.5%, *P* = 0.025). In addition, a more significant deterioration of eGFR, cystatin C, and SCr from 48 h to 1 week (*P* < 0.05) was observed in controls compared to the BNP group. Although eGFR gradually deteriorated in both groups, a faster recovery was achieved in the BNP group. Multivariate logistic regression revealed that using >100 mL of CM (odds ratio: 4.36, *P* = 0.004) and BNP administration (odds ratio: 0.21, *P* = 0.006) were independently associated with CIN. Combined with hydration, exogenous BNP administration before CM effectively decreases CIN incidence in CKD patients.

## 1. Introduction

Contrast-induced nephropathy (CIN) is a prevalent but underdiagnosed complication of percutaneous coronary intervention (PCI) and is associated with prolonged hospitalization and high mortality [1]. Although the exact pathogenesis of CIN is still incompletely understood, multiple factors, including renal vasoconstriction, direct cytotoxic effects of contrast media (CM), oxidative stress, inflammation, and tubular obstruction, are likely involved [2, 3]. Many risk factors are associated with CIN including the preexistence of renal dysfunction, hypotension, heart failure, diabetes mellitus, older age, anemia, and CM amount and type [4–6]. Among them, baseline renal dysfunction is arguably the most important risk factor [6]. Indeed, the incidence of acute renal insufficiency after PCI ranges from 2% in patients with normal baseline renal function to 20–30% in those with baseline serum creatinine (SCr) levels >176 *μ*mol/L (or >2 mg/dL) [7]. Therefore, protecting the kidneys before the use of CM is clinically relevant for patients with chronic kidney disease (CKD). Several prevention strategies have been proposed in recent years such as low dose of low osmolar or isotonic CM, hydration, and nephroprotective drugs (N-acetyl-cysteine, vasoactive drugs, and statins), but no optimal strategy for preventing CIN has yet been established.

After exposure to CM, an initial increase in blood flow occurs, followed by a sustained reduction due to a direct vasoconstrictor effect of CM, activation of the renin-angiotensin-aldosterone system (RAAS) and sympathetic nervous system (SNS), and CM-induced release of several endogenous vasoconstrictors such as adenosine and endothelin [2, 3, 8].

Brain natriuretic peptide (BNP) is a member of the natriuretic peptide family, has vasodilatory functions [9], and has various renoprotective effects (including renal plasma flow improvement, reduction of sodium reabsorption in the proximal tubule and collecting duct, and lowering of plasma levels of several vasoconstrictors) [10]. Studies have reported that low dose of atrial natriuretic peptide (the first member of the natriuretic peptide family) is beneficial in acute renal impairment after CM exposure [11, 12]. Interestingly, BNP infusion not only inhibits the systemic and regional (renal and cardiac) sympathetic tones [13] as well as the RAAS [14], but also decreases endothelin release [15]. Furthermore, BNP has multiple beneficial effects on renal function [16–18]. With respect to vasodilatation, BNP may increase renal blood flow and glomerular filtration rate [19].

Despite the large body of evidence regarding the beneficial effects of BNP, studies assessing this natriuretic peptide for its preventive role in CIN are scarce. Therefore, the present study aimed to determine whether low-dose BNP has a prophylactic effect against CIN in patients with CKD undergoing PCI or coronary angiography (CAG). We found that exogenous BNP administration before CM exposure significantly decreases CIN incidence in patients with CKD.

## 2. Patients and Methods

### 2.1. Study Design

This study was approved by the Ethics Committee of the Second Hospital of Hebei Medical University. All patients provided a written informed consent before enrolment. This was a randomized, open-label, control trial of rhBNP versus vehicle control. Patients were randomly assigned to one of the two groups in a 1 : 1 ratio in permuted blocks of four.

### 2.2. Patients

Consecutive eligible patients with CKD aged between 18 and 80 years undergoing CAG or elective PCI from October 2011 to October 2013 at the Second Hospital of Hebei Medical University were enrolled. CKD was defined as baseline estimated glomerular filtration rate (eGFR) between 15 and 60 mL/min/1.73 m^2^ as assessed by the simplified Modification of Diet in Renal Disease (MDRD) formula: eGFR = 186.3 (SCr)^−1.154^ (age)^−0.203^ (female: ×0.742) [20]. Patients with acute myocardial infarction (AMI) (ST segment as well as non-ST segment elevation MI) who needed emergency PCI were excluded. In addition, individuals with unstable angina who needed early invasive therapy (within 12–24 h) were not enrolled; they comprised patients with (1) refractory angina, or hemodynamic or electrical instability, (2) elevated risk of clinical events (heart failure/serious ventricular arrhythmias), and (3) high-risk (resting angina within 48 h or infarction angina, ST segment depression more than 1 mm and 20 min, and elevated cardiac biomarkers such as troponin [Tn] T and TnI). Other exclusion criteria were as follows: heart dysfunction (symptoms of dyspnea, orthopnea, or paroxysmal nocturnal dyspnea, accompanied by a left ventricular ejection fraction <40%); hypersensitivity to CM or BNP; end-stage renal failure; systolic blood pressure ≤100 mmHg before study drug infusion; CM administered within the past 7 days; BNP infusion within 1 month; administration of dopamine, N-acetyl-cysteine, sodium bicarbonate, and fenoldopam during the study.

Clinical data of all enrolled patients were obtained, including demographic data, medical history, laboratory results, medications, the extent of coronary artery lesions, and CM amount and type. Adverse events occurring during hospitalization and during the month after hospitalization were recorded. Hypotension was defined as systolic pressure <90 mmHg.

### 2.3. Procedure

Patients were randomized to the control (*n* = 103) or BNP (*n* = 106) groups. Patients in the BNP group received 0.005 *μ*g/kg/min of rhBNP (Lyophilized Recombinant Human Brain Natriuretic Peptide, Chengdu Nuodikang Biological Pharmaceutical Co. Ltd., China) for 24 h before the procedure (CAG or PCI) and hydration (0.9% NaCl at 1.0 mL/kg/h for 12 h before and 12 h after CM administration). Controls received hydration only. Randomization was performed at a 1 : 1 ratio with a computer-generated random number table. Visipaque (Iodixanol Injection, GE Healthcare, Ireland), a nonionic, isotonic CM, was used in all patients.

The primary endpoint was the incidence of CIN, which was defined as a relative (≥25%) or absolute (≥0.5 mg/dL, 44 *μ*mol/L) increase in SCr from baseline within 48 h after CM exposure. The secondary endpoints were the changes in SCr, eGFR, and serum cystatin C levels before and after the procedure. Cystatin C levels, SCr levels, and eGFR were assessed before and at 24 h, 48 h, 1 week, and 1 month after the procedure (CAG or PCI).

### 2.4. Detection of SCr Levels

Blood samples (3 mL) were collected in tubes containing an anticoagulant 24 h before angiography and BNP infusion and at 24 h, 72 h, 1 week, and 1 month after CM exposure. Patients were required to fast overnight before blood collection in the morning. Blood samples were centrifuged at 800 rpm and room temperature (20–25°C) for 10 min to obtain serum. SCr levels were determined by the picric acid method. Cystatin C levels were assessed by immunonephelometry on a Roche 8000 autoanalyzer (Roche, Switzerland).

### 2.5. Statistical Analysis

Sample size (*n*) was determined by the equation *n* = (*Z*_1_ − *α*/2 + *Z*_1_ − *β*)2 × [*P*_1_(1 − *P*_1_) + *P*_2_(1 − *P*_2_)]/(*P*_1_ − *P*_2_)2, with *α* = 0.05; *β* = 0.1; *P*_1_ = 16%, CIN incidence in the control group; *P*_2_ = 6%, CIN incidence in the BNP group. The modified intention-to-treat population was analyzed. All statistical analyses were performed using SPSS 13.0 (SPSS Inc., Chicago, IL, USA). Data are presented as mean ± standard deviation (SD) or median (interquartile ranges) for continuous variables and proportions for categorical variables. The independent samples *t*-test was used to compare continuous variables and the *χ* ^2^-test or Fisher's exact test for categorical variables. Repeated measure ANOVA and the Bonferroni post hoc test were used to evaluate the changes in variables within the same group. Multivariate logistic regression analysis was used to identify independent predictors of CIN. Two-sided *P* values <0.05 were considered statistically significant.

## 3. Results

### 3.1. Characteristics of the Patients

**Figure 1:**
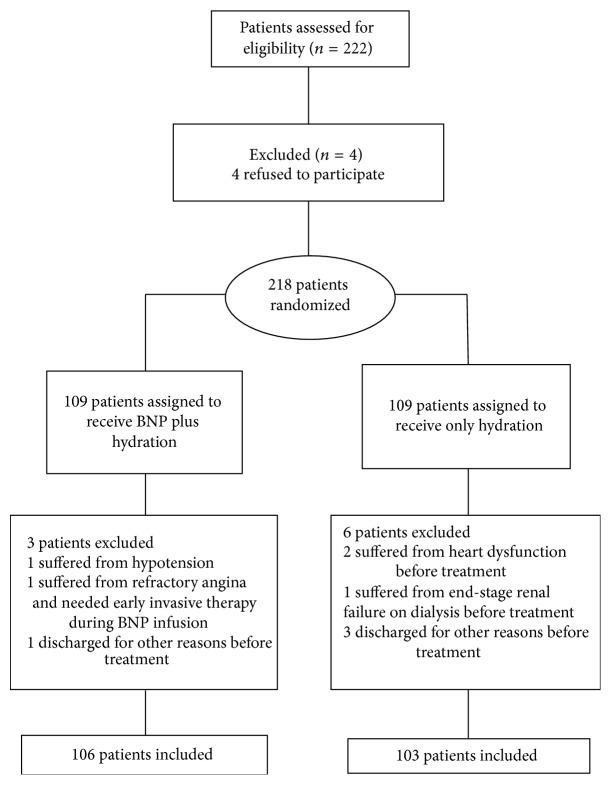
Patient flowchart. Hypotension: systolic pressure <90 mmHg.

A total of 222 patients were initially eligible for this study. Four declined to participate. Of the 218 randomized patients (BNP group, *n* = 109; control group, *n* = 109), 209 completed the study. Before starting the trial, one patient suffered from hypotension; two patients had heart dysfunction (symptoms of dyspnea, orthopnea, or paroxysmal nocturnal dyspnea, accompanied by a left ventricular ejection fraction <40%); one patient suffered from refractory angina and needed early invasive therapy before the study; one patient had end-stage renal failure; and four patients were lost to follow-up after the procedure. Finally, 106 and 103 patients were analyzed in the BNP and control groups, respectively (Figure 1).

The 209 patients included 133 men and 76 women, aged 68.7 ± 8.9 years; no significant differences between the two groups were observed for demographic data, medical history, laboratory test, medications, the extent of coronary artery lesions, and CM amount (Table 1).

### 3.2. Incidence of CIN

**Figure 2:**
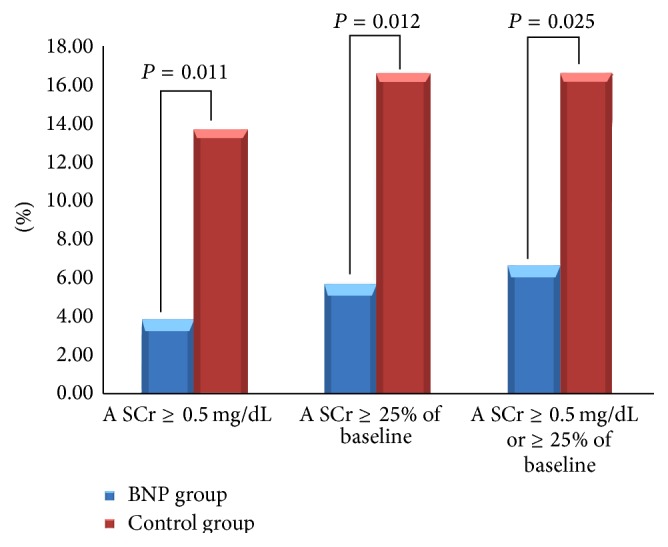
Incidence of CIN in the BNP and control groups according to different definitions of CIN.

A lower incidence of CIN was found in patients treated with rhBNP compared to controls. Indeed, the incidence of a SCr increase of ≥25% or ≥0.5 mg/dL from baseline was significantly lower in the BNP group compared to controls (6.6% versus 16.5%, *P* = 0.025). The incidence rates of a SCr increase of ≥0.5 mg/dL were 3.8% and 13.6% in BNP-treated patients and controls, respectively (*P* = 0.011). When defined as a SCr increase of ≥25%, the incidence of CIN was also lower in the BNP group compared to the control group (5.7% versus 16.5%, *P* = 0.012) (Figure 2).

### 3.3. Effects of rhBNP on Renal Function

As shown in Table 2, baseline cystatin C (1.14 ± 0.22 versus 1.17 ± 0.36, *P* = 0.251), SCr (117.2 ± 13.1 versus 120.5 ± 14.7, *P* = 0.33), and eGFR (52.3 ± 11.2 versus 50.9 ± 9.3, *P* = 0.293) were similar between the two groups. Nevertheless, a more significant deterioration was observed in the control group compared to BNP-treated patients. Cystatin C levels for the control and BNP groups were 1.56 ± 0.29 mg/L and 1.75 ± 0.94 mg/L (48 h, *P* = 0.027) and 1.20 ± 0.24 mg/L and 1.88 ± 0.82 mg/L (1 week, *P* = 0.006), respectively. Meanwhile, 140.1 ± 13.9 *μ*mol/L and 151.2 ± 15.9 *μ*mol/L were observed for SCr in the control and BNP groups at 48 h (*P* = 0.017), respectively, and 123.8 ± 14.4 *μ*mol/L and 159.7 ± 13.8 *μ*mol/L, respectively, at 1 week (*P* < 0.001). eGFR of 43.6 ± 17.1 mL/min and 40.2 ± 18.7 mL/min were observed in the control and BNP groups, respectively, at 48 h (*P* = 0.046) and 50.4 ± 14.9 mL/min and 37.9 ± 15.9 mL/min at 1 week (*P* < 0.001). There were no significant differences between the two groups for the mean eGFR, cystatin C, and SCr values at 1 month.

**Figure 3:**
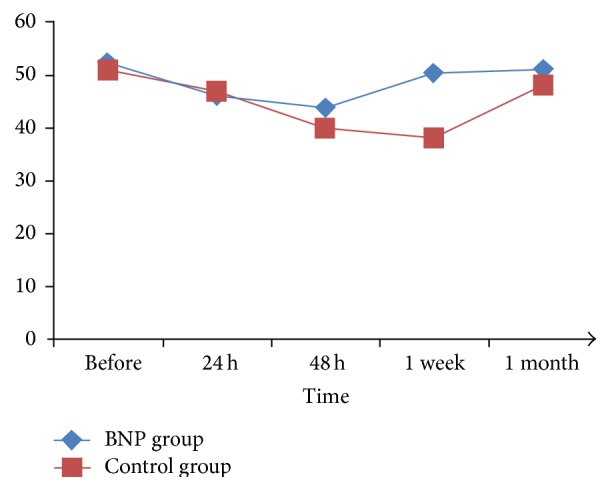
Changes of eGFR in the two groups after contrast media exposure.

Overall, eGFR gradually deteriorated until 1 week, followed by a mild improvement at 1 month in the control group, whereas the deterioration observed from 24 h to 48 h in the BNP group was completely restored at 1 week, and values remained at baseline until 1 month (Figure 3).

### 3.4. Adverse Effects

Besides the nine patients who suffered from complications before starting the trial, no patient presented adverse effects related to the use of rhBNP.

### 3.5. Subgroup Analysis

The predictors of CIN were explored. Univariate analyses showed that patients who developed CIN were more likely to be older (71.4 versus 62.1 years, *P* = 0.023), to have a history of diabetes (62.3% versus 35.8%, *P* = 0.011), to have hypertension (36.9% versus 22.4%, *P* = 0.031), to have received PCI (*P* = 0.014), to have received more CM (158 versus 78 mL, *P* = 0.002), and to be non-BNP-treated (*P* < 0.001). After multivariate logistic regression analysis, only the CM volume (CM >100 mL) and BNP administration were significant independent predictors of CIN (Table 3).

## 4. Discussion

The results of this study showed that a more significant deterioration of eGFR, cystatin C, and SCr was observed in the control group compared to BNP-treated patients. Consequently, a lower incidence of CIN was observed in patients to whom rhBNP was administered. These findings strongly suggest that the prophylactic use of BNP before PCI or CAG may help prevent CIN.

CIN is commonly defined as a relative (≥25%) or absolute (≥0.5 mg/dL) increase in SCr levels from baseline. Basically, SCr rises within the first 24–48 hours after CM exposure, peaks at 3–5 days, and returns near baseline values within 1–3 weeks [21]. However, the SCr peak is postponed in patients with preexisting impaired renal function, and the increase may last for 7–21 days [2]. In this study, SCr, eGFR, and cystatin C levels were deteriorated until 1 week after the procedure, before returning to baseline at 1 month in the control group. In contrast, a less pronounced deterioration of these kidney function markers was achieved in BNP-treated individuals, with a nadir observed at 48 h and complete restoration to baseline levels by 1 week after the procedure. These findings indicate that BNP administration may have beneficial effects on renal function recovery after CM exposure. Among other functions described previously [13–15], BNP was shown to positively affect the renal function [16–18], for example, increasing renal blood flow and glomerular filtration rate [19].

Apart from BNP administration, CM volume >100 mL was found to be an independent predictor of CIN in this study. This finding is supported by previous reports demonstrating that large CM volumes are associated with an increased risk of CIN [8, 22, 23]. Although no definitive threshold values have yet been established, CM volumes ≥100–200 mL are associated with a higher incidence of CIN in high-risk patients [24]. It was found that, in patients undergoing PCI, each 100 mL of CM is associated with a 12% increased risk of CI-AKI [7]. Taken together, CM volume should be limited as much as possible in high-risk patients.

A few limitations of the present study should be mentioned. First, the relatively small sample size prevents the generalization of our findings. In addition, this was a single center trial with the inherent selection bias. Finally, the study was not blinded. Therefore, multicenter, randomized, controlled trials with a larger sample size are required to further evaluate the beneficial effects of BNP on CIN.

## 5. Conclusions

Overall, this study showed that, in addition to hydration, exogenous administration of BNP before CM exposure is effective in decreasing the incidence of CIN in patients with CKD.

## Figures and Tables

**Table 1:** Baseline characteristics of the patients.

Variables	Control group (−) (*n* = 103)	BNP group (+) (*n* = 106)	*P*
Sex (male), *n* (%)	63 (61.2%)	70 (66.0%)	0.464
Age (years)	69.8 ± 6.7	67.6 ± 7.2	0.512
Body mass index (kg/m^2^)	25.4 ± 4.2	24.9 ± 5.0	0.419
SBP	133.4 ± 14.6	138.8 ± 13.9	0.187
Baseline serum creatinine (*μ*mol/L)	120.5 ± 14.7	117.2 ± 13.1	0.206
Estimated glomerular filtration rate (mL/min)	50.9 ± 9.3	52.3 ± 11.2	0.324
Cystatin C	1.17 ± 0.36	1.14 ± 0.22	0.275
History of diabetes mellitus, *n* (%)	71 (68.9%)	76 (71.7%)	0.662
History of hypertension, *n* (%)	59 (57.3%)	62 (58.5%)	0.860
Left ventricular ejection fraction, *n* (%)	58.4 ± 10.5	61.1 ± 8.2	0.337
Isosorbide dinitrate, *n* (%)	77 (74.8%)	75 (70.8%)	0.516
Low molecular heparin, *n* (%)	98 (95.1%)	100 (94.3%)	0.794
Statins, *n* (%)	102 (99%)	103 (97%)	0.622
Contrast volume (mL)	96 ± 14.5	102 ± 17.2	0.318
Contrast volume >100 mL, *n* (%)	62 (60.2%)	65 (61.3%)	0.757
Single-vessel disease, *n* (%)	35 (34%)	37 (34.9%)	0.888
Double-vessel disease, *n* (%)	42 (40.8%)	46 (43.4%)	0.701
Three-vessel disease, *n* (%)	26 (25.2%)	23 (21.7%)	0.545
Coronary angiography, *n* (%)	36 (35%)	33 (31.1%)	0.557
PCI, *n* (%)	67 (65%)	73 (68.9%)	0.557

Note: continuous variables are expressed as mean ± standard deviation. Categorical variables are presented as percentage. SBP: systolic blood pressure.

**Table 2:** Changes of renal function before and after the procedure between the two groups.

Group	Cystatin C (mg/L)	SCr (*μ*mol/L)	eGFR (mL/min)
BNP group (*n* = 106)			
Baseline	1.14 ± 0.22	117.2 ± 13.1	52.3 ± 11.2
After procedure			
24 h	1.44 ± 0.31^#^	133.2 ± 14.1^#^	46.3 ± 15.4^#^
48 h	1.56 ± 0.29^#^	140.1 ± 13.9^#^	43.6 ± 17.1^#^
1 week	1.20 ± 0.24	123.8 ± 14.4	50.4 ± 14.9
1 month	1.16 ± 0.20	120.7 ± 15.1	50.9 ± 18.2
Control group (*n* = 103)			
Baseline	1.17 ± 0.36	120.5 ± 14.7	50.9 ± 9.3
After procedure			
24 h	1.51 ± 0.44^#^	137.4 ± 14.1^#^	46.8 ± 12.6
48 h	1.75 ± 0.94^*∗*#^	151.2 ± 15.9^*∗*#^	40.2 ± 18.7^*∗*#^
1 week	1.88 ± 0.82^*∗*#^	159.7 ± 13.8^*∗*#^	37.9 ± 15.9^*∗*#^
1 month	1.19 ± 0.26	129.6 ± 14.6	48.2 ± 15.7

SCr: serum creatinine; eGFR: estimated glomerular filtration rate.

^*∗*^ *P* < 0.05 versus the BNP group (data were analyzed using the independent samples *t*-test). ^#^ *P* < 0.05 versus baseline within the same group (data were analyzed using repeated measure ANOVA and the Bonferroni post hoc test).

**Table 3:** Univariate and multivariate analyses of CIN predictors.

Variables	Univariate odds ratio (95% CI)	*P* value	Multivariate odds ratio (95% CI)	*P* value
Age	3.08 (2.23, 4.29)	0.023^*∗*^	0.86 (0.62, 0.98)	0.344
Diabetes mellitus	4.71 (4.04, 6.13)	0.011^*∗*^	2.12 (1.08, 3.66)	0.083
Hypertension	2.96 (1.75, 5.17)	0.031^*∗*^	1.13 (0.61, 2.07)	0.184
Contrast volume >100 mL	5.32 (4.13, 6.65)	0.002^*∗*^	4.36 (2.23, 5.47)	0.004^*∗*^
Type of procedure	3.48 (2.06, 4.12)	0.014^*∗*^	1.04 (0.61, 4.14)	0.321
BNP administration	6.27 (4.46, 8.23)	<0.001^*∗*^	0.21 (0.09, 0.46)	0.006^*∗*^

^*∗*^ *P* < 0.05.
